# Learning to BREATHE “Plus”: A Multi-Modal Adaptive Supplement to an Evidence-Based Mindfulness Intervention for Adolescents

**DOI:** 10.3389/fpubh.2020.579556

**Published:** 2020-11-17

**Authors:** Rachel G. Lucas-Thompson, Stephanie Rayburn, Natasha S. Seiter, Patricia C. Broderick, Joshua M. Smyth, J. Douglas Coatsworth, Kimberly L. Henry

**Affiliations:** ^1^Department of Human Development and Family Studies, Colorado State University, Fort Collins, CO, United States; ^2^Bennett-Pierce Prevention Research Center, Pennsylvania State University, University Park, PA, United States; ^3^Biobehavioral Health and Medicine, Pennsylvania State University, University Park, PA, United States; ^4^Department of Psychology, Colorado State University, Fort Collins, CO, United States

**Keywords:** adolescence, mindfulness-based interventions, ecological momentary intervention, ecological momentary assessment, home practice

## Abstract

Incorporating technological supplements into existing group mindfulness-based interventions (MBIs), particularly for use with adolescents, is an important next step in the implementation of MBIs. Yet there is little available content. Herein we present the development and content of a technological supplement for MBIs, which incorporates multiple technological elements to support (a) skill transfer from the group MBI to daily life, (b) the establishment of a formal mindfulness practice, and (c) the use of mindfulness during periods of high stress. A mixed-methods approach was used to develop this multi-method adaptive supplement. Findings about the use of this supplement will be disseminated scientifically and/or publicly as appropriate.

## Introduction

Mindfulness-based interventions (MBIs) provide participants with opportunities to practice cultivating attention to the present moment with non-judgment, and have a robust and growing evidence base to support their use, particularly for adults [e.g., (1–4)]. MBIs are also well-liked by and effective for adolescents, and improve not only mindfulness but also emotion regulation and coping, as well as decrease internalizing symptoms, externalizing symptoms, and sleep problems (5–12).

Empirical studies have begun to explore stand-alone brief and online MBIs [e.g., (13)], and suggest that they can be effective [e.g., (14)]. However, to our knowledge, no studies have evaluated technological supplements to an in-person MBI. We have argued elsewhere that, particularly when working to increase mindfulness in adolescence, a critical next step in MBI implementation science is to utilize a mobile-technology-enhanced MBI; i.e., to incorporate technological supplements into an existing MBI (15). In the current paper, we detail the development and content of just such a mobile-technology-enhanced MBI to promote mindfulness in adolescent populations.

## The Case for Incorporating Technology Into an MBI for Adolescents

Over the last several decades, mental health problems (16), and levels of stress (17) during adolescence have risen dramatically, such that adolescents now report levels of stress that are similar to those reported by adults (17). Although there are fewer studies evaluating the effects of MBI on adolescents than those evaluating the effects on adults, work focusing on adolescents indicates that MBIs improve mental health in both clinical and non-clinical samples, relative to active controls (18), and with effects that persist beyond program cessation (19).

A critical part of MBIs is encouraging participants to transfer skills learned in the program into their daily life, with emphasis on the necessity of a regular mindfulness practice in order to experience benefits for well-being [e.g., (20)]. This emphasis is supported by empirical work which suggests that the time spent practicing mindfulness between in-person sessions predicts improvements in both mindfulness and mental health (21). Furthermore, associations between mindfulness practice time and subsequent mental health are mediated by increases in mindfulness (21). However, adolescent participants in MBIs typically have very low levels of compliance with home practice recommendations. For instance, on average, adolescents engage in home practice on only about ¼ of the days they are encouraged to by intervention facilitators (22).

Based on this research, there appears to be a need to increase home practice and skill transfer for adolescents participating in MBIs. An accompanying body of literature suggests that technological tools are a very effective way to support skill transfer, particularly for adolescents. In particular, mobile technology (i.e., cell phones) has become widely incorporated into adolescent daily life (23). Most (i.e., >78%) of adolescents own mobile phones (23), with rates of cell phone ownership that are relatively equal across ethnic and socioeconomic groups (23, 24). Adolescents communicate more frequently over text than face-to-face and also endorse text as their preferred method of communication, sending a median of 60 text messages a day (25, 26). Therefore, not only is the ownership of mobile phones among adolescents almost ubiquitous, there is a seamless integration of mobile devices into the lives of adolescents. Therefore, intervention content (structured or unstructured) can be delivered via mobile technology in ecologically valid ways, in people's daily lives and natural settings. Such approaches are called ecological momentary interventions, or EMI (27). Interventions that incorporate EMI may be more likely to result in lasting behavioral and mental health change for adolescents (27, 28) because they are well-liked by adolescents (28–30), are easily assimilated into daily life for adolescents (28), and strongly support skill transfer by offering intervention content during daily life when adolescents are more likely to apply their developing skills (27). In addition, EMI components are highly flexible, and can be delivered on a pre-programmed schedule or in response to real-time participant data reflecting moments or high need (31). As such, there is a clear opportunity to leverage technology as a means of “meeting teens where they are” as digital natives to enhance engagement and home practice, in order to improve outcomes for adolescents.

Although EMIs can be stand-alone interventions or supplements to existing intervention strategies (27), there are multiple lines of evidence that converge to suggest that it may be optimal to combine EMI elements with other mindfulness intervention elements. Notably, EMI are often more efficacious when they are combined with other treatment strategies (e.g., in-person and/or group treatment) rather than when used as a stand-alone intervention (27). For instance, research in education suggests that EMI supplements enhance the effect of in-person educational interventions (32), though EMI supplements have not been tested in mindfulness interventions. Furthermore, many scholars and practitioners argue that the interpersonal and group dynamic of in-person MBIs are a very important part of the intervention [e.g., (20)]. However, studies examining technological approaches to increasing mindfulness have almost exclusively focused on stand-alone MBIs. These studies indicate that MBIs delivered online, through self-help or individually guided learning, or through a smartphone application, can increase mindfulness and lower psychological symptoms (13, 14, 33–38). Therefore, despite potential challenges of incorporating technology into an MBI [for a review, see (15)], we argue that an important next step in the implementation of MBIs is to incorporate an EMI, particularly when working to increase adolescent mindfulness. In the current paper, we discuss the development and content of a multi-method, adaptive supplement to an evidence-based MBI for adolescents.

## Learning to Breathe: The Adolescent MBI to Supplement

Our focus on adolescence meant it was important for us to select a developmentally appropriate MBI as the foundation for an EMI supplement. In addition to increases in mental health problems during adolescence (16), the challenges of treating adolescents' mental health problems may be due in part to the use of treatment strategies that have not adequately taken into account the unique developmental characteristics of adolescence (39). There is growing evidence to support the effectiveness of Learning to BREATHE (L2B) (6), an MBI that is rooted in the philosophy of mindfulness-based stress reduction (MBSR; 20) but developed to be developmentally sensitive to the unique characteristics of adolescence (40). The program was developed based on the same meditative training tradition approach used in MBSR (practicing cultivating attention that is purposeful, present-focused, open and non-reactive), as well as the based on the same three families of practices: focused attention (e.g., awareness of breath), open awareness (i.e., awareness of bodily sensations, thoughts, and feelings as they occur), and compassion (i.e., loving kindness and compassion for self and others). However, L2B was further tailored to: (1) support adolescent empowerment, autonomy, and self-efficacy in the face of stress; (2) build adolescent skills for emotion regulation, a key developmental task of adolescence; (3) encourage group cohesion by focusing on the common experiences of adolescents; (4) reduce tendencies for social comparison and self-judgment; and, (5) encourage peer acceptance and support via shared practice and activities. Therefore, it is a developmentally sensitive MBI. In addition, L2B is well-liked by adolescents and increases mindfulness as well as emotion regulation while it reduces stress, internalizing symptoms, and externalizing behaviors (5–12).

L2B is built to support practice and learning of six core themes built around the acronym BREATHE: Body, Reflections, Emotions, Attention, Tenderness, and Habits, all building to the overall program goal of Empowerment. These themes can be delivered in 6, 12, or 18 sessions, and in schools or in the community [see (41) for more details]. For the purposes of our work, we have implemented the 6-session version of L2B, although given the overlap in progression and content between the 6, 12, and 18 session programs, and the flexibility of the “Plus” component, “Plus” could effectively be used to support the longer programs as well, with relatively minor modifications. In this paper, we describe the combined intervention strategy of in-person L2B plus the multi-method adaptive supplement that we designed, or L2B Plus.

## Elements of L2B Plus

The philosophy guiding the development of L2B Plus was to supplement the L2B group program with multiple methods of support for practicing mindfulness in daily life and particularly during times of high need, in ways that augment and are consistent with each L2B theme/lesson. We developed multiple methods to accomplish the interconnected goals: (1) support for establishing a formal mindfulness practice; (2) support for practicing formal and informal mindfulness practices in daily life, and (3) support for using mindfulness in moments of high need (which requires first identifying those moments when support is highly needed).

Adolescents participating in L2B are encouraged to engage in practices that are both formal (intentional time set aside for a mindfulness practice; those included in L2B are similar to those for adults such as a body scan, but often of a shorter length, as is more appropriate for adolescents) and informal (e.g., paying attention to breathing throughout the day, noticing sensations in the body when emotions become dysregulated). Both formal and informal practices are important: for instance, for adults, time spent in formal (but not informal) mindfulness practice predicts increases in mindfulness as well as improvements in mental health (21), whereas informal practices are believed to be critical for transferring the skills from formal practices into real life (20). When individuals are first developing a mindfulness practice, it is typical and helpful to follow guided mindfulness practices.

First, to support participants establishing a formal mindfulness practice, we developed an extensive on-demand library of educational materials and guided mindfulness practices, consistent with the content of L2B. In L2B, like other MBIs, brief didactic teachings are used to help adolescents learn about mindfulness and its benefits and are intended to help participants apply these skills in their lives. To extend and deepen these teachings, we included supplemental, developmentally appropriate education about mindfulness in our extensive on-demand library.

Second, we developed a set of messages that can be sent to adolescents (via text message or push notifications) to support practicing mindfulness in daily life both formally and informally (i.e., an intervention text message bank). Using previous theory and evidence, we developed three different types of text messages (reminders, motivational, and self-efficacy). As noted, adolescent compliance with home mindfulness practice recommendations is generally poor (22); it is unclear, however, what specific roadblocks to home practice adolescents experience. Some anecdotal data from our own L2B facilitation suggests that one important roadblock to developing a regular mindfulness practice is remembering to practice. For instance, at the beginning of each L2B meeting after the first, adolescents are invited to share things that have been going well and things that have been challenging in terms of practicing mindfulness at home. Adolescents often share that they forgot to practice, and/or what exactly they were supposed to be practicing. Perhaps not surprisingly, then, many report appreciating a homework assignment called “three dots” in which they are provided with three colored stickers, and instructed to place them in visible places. Then, each time they see a dot, they are instructed to take three mindful breaths. Therefore, the first category of messages we developed to support the development of a regular mindfulness practice was *reminders* about what adolescents learned in that week's lesson and what would be beneficial to practice.

The broader literature on behavior change suggests several other possible challenges to the process of beginning to incorporate mindfulness into daily life, namely low levels of motivation and/or self-efficacy (i.e., beliefs about one's ability to meet a goal or engage in a behavior). For instance, the information-motivation-behavioral skills (IMB) model, developed in relation to HIV risk behaviors (42) but much more widely applied in behavior change work (43), highlights issues of motivation and self-efficacy as modifiable factors that are central to creating and maintaining behavior change. Therefore, the second category of messages that were developed were *motivational*, or messages that reinforce the importance of developing a mindfulness practice (i.e., in what ways increasing mindfulness can be beneficial). In keeping with the IMB model, as well as extensive and consistent evidence that self-efficacy is a one of the strongest predictors of behavior change (44, 45), our third category of messages targeted *increasing self-efficacy*. These messages were positively framed, in keeping with evidence about what types of messages are most effective at increasing self-efficacy (46). Therefore, they focused on emphasizing that adolescents already had the skills and/or attributes to successfully establish a mindfulness practice, and how to expand upon those newly developing skills to establish other new, desirable habits.

Our third goal was to support using mindfulness particularly in moments of high need, which necessitates first identifying those moments of high need. The key mental and physical health benefits of mindfulness are theoretically rooted in its ability to buffer individuals from the negative consequences of stressful experiences (47). Therefore, we conceptualized “high need” as times of high stress (broadly defined) and/or low mindfulness. As a result, a key element of L2B Plus is ecological momentary assessments (EMA) that are used throughout the day to ask participants to report on their levels of stress/mindfulness so that intervention content can be delivered “just-in-time” (JIT) to real-time data from participants. These JIT messages were intended to support adolescents applying or using mindfulness (including self-compassion) during periods of high stress.

## Methods

### Creation of the On-Demand Library

Elsewhere, we provided a brief overview of the methods used to develop the on-demand library (48). Here, we provide further details about this development and fuller description of the content of this library. To develop the extensive on-demand library, we (1) conducted a thorough search of freely available online mindfulness education and practices using general mindfulness terms as well as keywords from L2B themes and major practices, and (2) identified gaps in this content and created new content to fill those gaps. Both the selection of existing and creation of new content followed the same basic decision rules and/or exclusion criteria. Content was selected if it did not include any of the following exclusion criteria, and was created to avoid each of the following: (1) technical problems (e.g., with sound quality); (2) free use not allowed; (3) explicit religious reference (in either language or imagery); (4) content that was not developmentally appropriate for adolescents; (5) length of >20 min; (6) unclear or confusing practice instructions (e.g., instructions that were contradictory or vague); (7) pedagogically inappropriate as a supplement to L2B (i.e., not reflective of L2B themes of practices taught in the group program; and, (8) inappropriate types of mental training (i.e., mantra meditations, visualizations, analytical meditation, relaxation training) that may have been very high-quality but are not part of L2B. In terms of this last criteria, as noted, L2B (like the meditative tradition used in MBSR) relies on three families of practices: focused attention, open awareness, and compassion practices. Other types of contemplative practices are important in other practice systems, and share similar goals with L2B/MBSR, but are not included in L2B. Therefore, they were also excluded from the “Plus” components, in order to reinforce what is taught in the group program and also to avoid confusion for participants. Finally, we identified and developed content that varied in length to provide participants with options that would be feasible for them to complete in a variety of situations and amounts of time.

Once content was identified and/or developed, the final step was to assign that content to the most appropriate L2B theme. This sorting was guided by the principle that students should have opportunities to independently (and in their daily life) practice skills that had already been introduced and practiced in the group program. In addition, in L2B, practices are scaffolded in a developmentally appropriate sequence. Therefore, the sorting of the on-demand library was completed in ways that provided students with practices that would match participants' level of understanding and ability (e.g., making sure concepts and techniques discussed in each practice did not go beyond what had already been introduced in class). As discussed in detail in the “Results” section, as with the progression of L2B, in the on-demand library, the practices that are available to participants in early weeks are limited primarily to focused attention on one object of attention (e.g., on breath or body), but in later sessions, participants focus on multiple objects of attention (e.g., body, thoughts, and feelings simultaneously).

### Creation of the Intervention Message Bank

To create the intervention message bank (as well as inform timing of message delivery), 22 adolescents (3 cohorts of 3–13 members aged 12–18) participated in the full L2B program, and then at the end of each weekly session, participated in an activity that was designed to help with the development of this message bank. Written informed consent was obtained from parents as well as adolescents 17 years of age and older; written informed assent was obtained from all other adolescents. More specifically, at the end of each session, each adolescent was asked to work independently to write down one or two short sentences that could serve as reminders and/or encouragements to practice mindfulness during the week, based on the content of that week's session. In addition, at the end of the group program, they participated in a focus group to assess issues related to the timing of message delivery (e.g., times of day that would be helpful and not helpful to receive messages).

The study team carefully reviewed the pool of potential intervention messages to select and/or modify those that were the most in line with the goals of each particular session. The study team then developed additional messages to cover the most important themes of each individual lesson, making sure that each theme was represented in the categories of reminders, motivational, and self-efficacy messages (one-third of developed messages were from each category). Each topic of L2B was represented in the text messages with the exception of “habits,” as this lesson is the last day of the program and therefore participants do not receive EMI content following this session. To select which specific elements from each lesson to include in individual messages, we first identified the general theme of each session (taken from the L2B session manuals) and the main practice of the session, as well as practices secondary and/or tertiary practices (typically designed to support the main practice), if they were included in the session. As an example, the general message of the “A” lesson is, “Attention to body, thoughts, and feelings is good stress reduction;” the main practice of this session is mindful movement/mindful walking, the secondary practice is an activity to learn about stress, and the tertiary practice was a psychoeducational lesson regarding the interconnection of body, thoughts, and feelings. These essential messages as well as main, secondary, and tertiary practices were used to frame the messages in the message bank.

### Creation of Just-in-Time Messages

Finally, the study team worked to create messages that were congruent with each L2B theme that would support practicing mindfulness in the face of stressful experiences. Messages were designed to be brief and general, so that they were applicable to a wide variety of possible unpleasant emotions and/or stressful experiences. These messages were intended to be stand-alone reminders about how to remain mindful even when feeling upset, stressed, or overwhelmed, so that participants might be better equipped to use mindfulness during moments of high need. Messages were framed compassionately, in line with the lessons of L2B. Each message also included links to two practices from the on-demand library that the study team identified as being helpful for enhancing mindfulness when under stress. Participants were given the opportunity to select one of the practices if interested and able to listen to one while they were struggling. One selected practice was very brief, and one was longer, to account for differences in the amount of time participants might have to engage in a practice.

## Results

### Overview

As our “Plus” components were designed to supplement the L2B group program (rather than to stand alone as a program), it is important to understand how the in-person and EMI elements are designed to fit together. An overview of the integration between the group program, on-demand library, intervention content messages, and EMA/JIT messages is provided in Table 1. As is evident in that table, after each week's content is introduced and practiced in the in-person group meeting, adolescents are given access to that week's content in the on-demand library (and are still given access to all of the previous weeks of content as well). The on-demand library also includes a recorded summary of each group meeting, so that students who miss a week can to some extent “catch up” on the material they missed. The day after the group meeting, adolescents also start receiving intervention content messages, as well as EMA to assess levels of stress and mindfulness, which can trigger the delivery of messages specifically designed to support remaining mindful when it is challenging. Both intervention content and JIT messages are tailored to that specific week of content (i.e., they focus on the theme discussed in the last week's group program). In the following sections, we provide more detail about each of the three important “Plus” components.

**Table 1:**
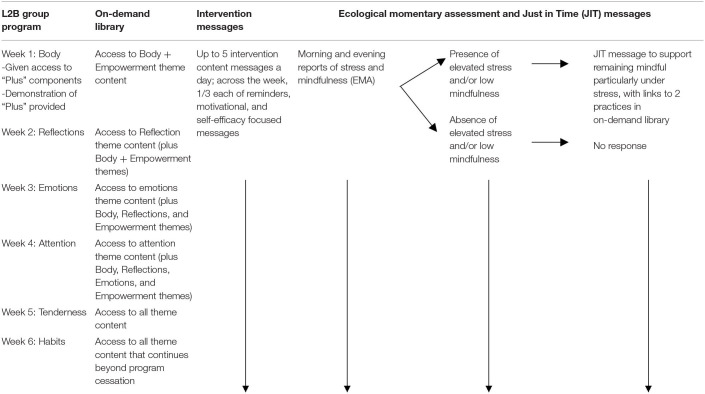
Integration of L2B with plus components.

### On-Demand Library

**Figure 1:**
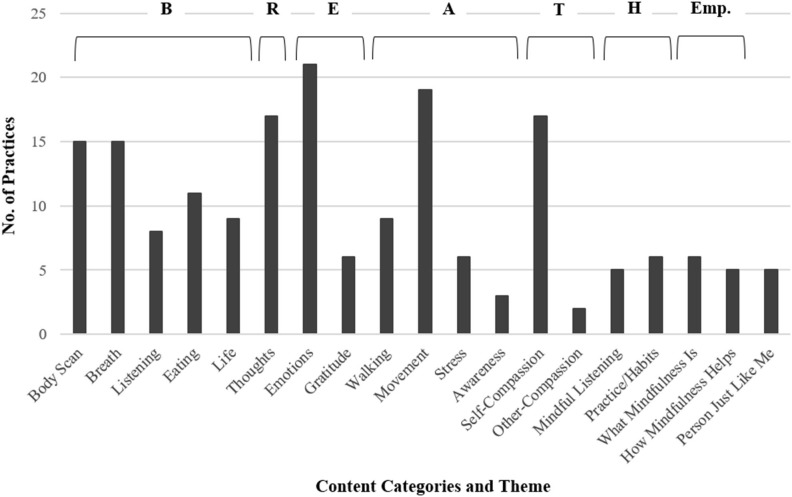
Specific practices included in the on-demand library for each theme of L2B. Some types of practices are included in small amounts in multiple week of L2B. Where that is the case, the practices here have been grouped with the theme where most are included. “Person Just Like Me” is a compassion practice considered to be a core component of L2B. One “Person Just Like Me” practice occurs in each week of L2B, so it has not been grouped into a single theme.

The on-demand library is a searchable database organized by weekly theme for the intervention. Themes become available as participants move through the in-person class sessions. For example, after the first session, participants are given access to the “B” theme and to the “Empowerment” theme. After the second session, participants are additionally given access to the “R” theme, and so on. After the last session, participants have access to all themes in the library. Content for each theme is targeted to the topics covered in class. See Figure 1 for information about the categories and quantity of content available for each theme.

**Figure 2:**
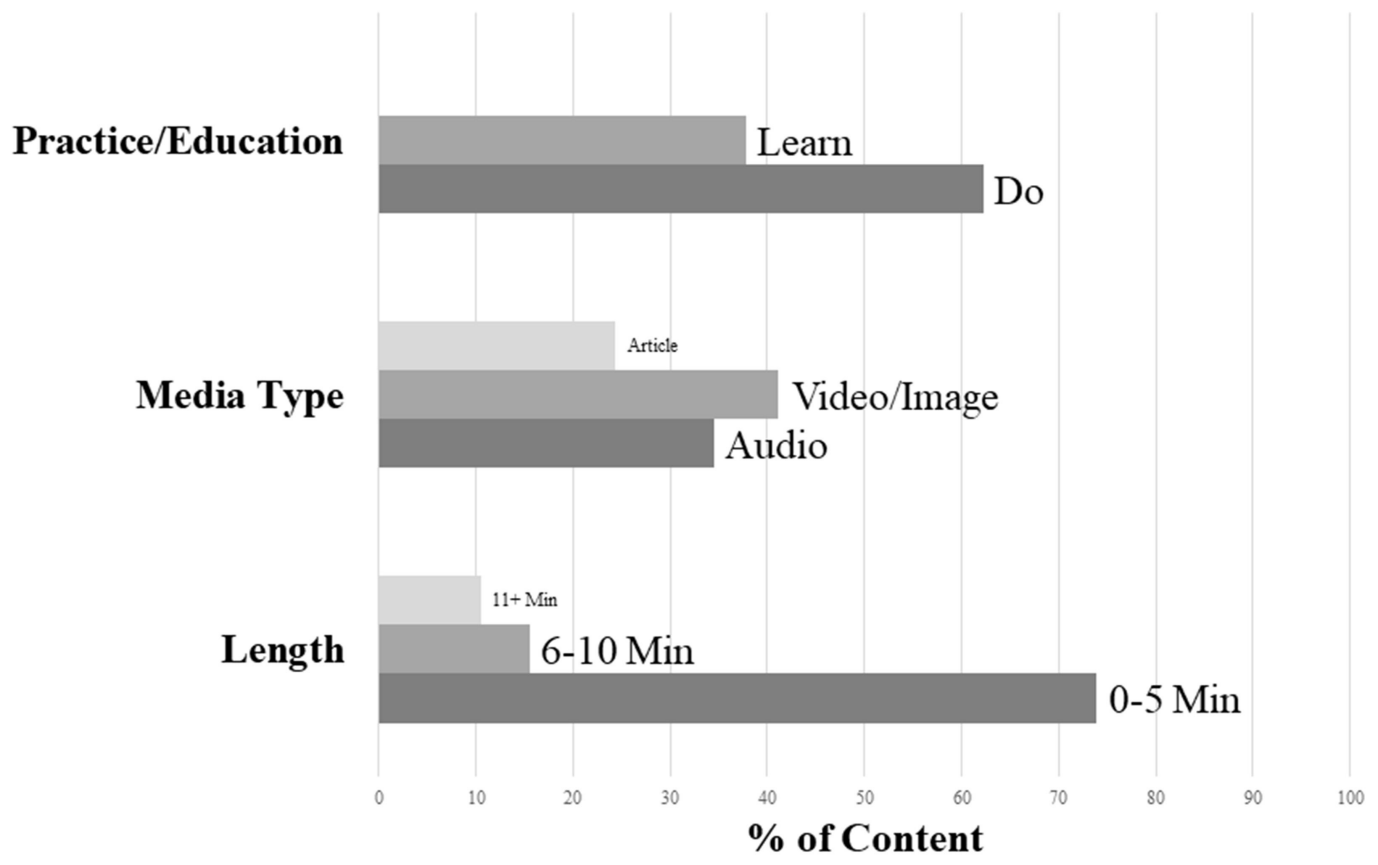
Percentages of on-demand library content types by practice vs. education, length, and media type.

In light of evidence that user-centered digital interventions promote greater engagement (49), we allowed participants to self-tailor their content to promote greater practice. Participants are able to search the available library by (a) theme, (b) length of practice (0–5, 6–10, 11+min, and up to 20 min), and (c) type of activity (e.g., watch an educational video about mindfulness, do a listening audio practice, read more about mindfulness, follow a written practice, watch a mindfulness practice video). Participants can also rate activities and/or save their favorites to facilitate returning to them in the future. In addition to searching the database of activities, each week, the home page is also updated with a few recommended practices for the current theme, allowing participants to quickly find practices and instructional content related to their most recent class. The on-demand library provides a blend of audio, video/image, and reading content. The library also emphasizes brief practices (<20 min and particularly <5 min), in keeping with feedback from adolescents about how much time they are willing to practice at any given time, as well as the length of practices introduced in the group program. See Figure 2 for information about the distribution of lengths, media format, and practice or education type for library content across weeks.

### Intervention Content Messages

We designed intervention content messages to be sent several times a day to participants (see Table 2 for example intervention text messages of each type for each message). These messages are also designed to be distributed flexibly, based on the needs of individual samples, and to allow for adolescent self-tailoring of distribution times. Messages have been developed to send up to five intervention text messages every day; for instance, first thing in the morning (7:00 am), before school (9:00 am), at lunchtime (12:00 pm), right after school (4:00 pm), at dinnertime (6:00 pm), or at bedtime (9:00 pm). The specific timing of these messages was based on focus groups conducted with the 22 adolescents who participated in the full L2B program; these times were reported as being appropriate and helpful times to receive intervention messages. Message delivery is distributed throughout the week by message type (reminder, motivational, or self-efficacy) and key theme (general theme, main, second, or tertiary practice) following a set pattern. Reminder and self-efficacy message types are sent at different frequencies throughout the week, with reminders being sent most frequently earlier in the week and self-efficacy sent later in the week. Motivational messages are sent at a similar and consistent frequency throughout the week. This method was chosen in an effort to support the development of knowledge retention earlier in the week, and then new ideas for expanding newfound skills later in the week. It is our belief that participants must first focus on *what* mindfulness is and *how* to practice it (the content of reminder text messages), and then be supported to build confidence that they can increase their mindfulness (the content of self-efficacy messages), with information about *why* to practice mindfulness (in motivational messages) spread throughout the week. In addition, messages regarding main practices and essential themes are weighted more heavily toward the beginning of the week as a way of solidifying the most important ideas from the last session before moving on to a higher concentration of messages about secondary and tertiary practices toward the end of the week.

**Table 2:** Example intervention content and just-in-time messages.

**Category**	**B**	**R**	**E**	**A**	**T**
Reminder	Reminder: You can bring your attention back to the present moment any time by focusing on your body or your breath	Reminder: The mind constantly chatters. We can work with the chattering mind by paying attention to thoughts and watching them come and go. We don't need to believe everything we think	Reminder: Emotions come and go like ocean waves. Even intense emotions will subside	Reminder: Attention to body, thoughts, and feelings is good stress reduction	Reminder: We sometimes get down on ourselves about the kinds of thoughts or feelings we have, or when we are feeling stressed or overwhelmed. Remember everyone sometimes struggles with difficult thoughts or emotions, and everyone is over-stressed at times. This is a normal part of being human, and we can have compassion for those experiences in ourselves and others
Self-efficacy	You can do any activity mindfully by using the skills you've been learning of paying attention, noticing when you attention wanders, and bringing it back to what you're doing!	Now that you are able to observe the stories your mind is creating without believing them or following them, you can use this skill when you notice you are having unpleasant thoughts, and say to yourself, “it's just my mind storytelling”	Now that you know how to surf the waves of your emotions, when you notice you are feeling a difficult emotion, you can feel the emotion mindfully by noticing it with curiosity and non-judgement	You have a great tool available to you when you are feeling stressed or anxious – try practicing a simple stretch like the palm-press or seated tree to come into the present moment and breathe into your experience. You can find a variety of seated and standing movement practices on the L2B+ website	You have the tools to be kind to yourself as you practice and form new healthy habits
Motivational	Try breathing out of your belly to relax! Sometimes breathing in and out of the chest can make us feel stressed or worried, but belly breathing relaxes us	Becoming more aware of your thoughts and accepting them as mental activity can help you both in daily life and when you are facing challenges	If you're feeling something unpleasant, you can think of one or two things you're grateful for to cultivate helpful emotions	Practicing mindfulness in daily life can help us to cope with the ups and downs of life in more healthy ways. This builds inner strength	Just as having judgmental thoughts about ourselves can increase our stress, having kind or compassionate thoughts about ourselves can reduce our stress
**“Just in Time” messages**					
N/A	Seems like you're going through a tough time. Try a quick body scan, feeling the sensations in your feet, belly, chest, and face. Here are a few practices that could help [link to 3-min body scan audio practice] [link to 9-min body scan audio]	You may be struggling right now. Remember that you don't have to believe everything you think; thoughts are just thoughts. Here are a few practices that could help [link to 1-min mindfulness of thoughts audio] [link to 14-min mindfulness of thoughts audio]	We don't have to cover up our emotions or avoid them. Give yourself permission to feel how you feel- your emotions are a part of your experience that you can be open to and allow. Here are a few practices that could help [link to “welcoming your emotions” image] [link to 5-min mindfulness of emotions audio]	You're having to handle a lot right now. Paying mindful attention to your body, thoughts, and feelings can help reduce stress. Here are a few practices that could help. [link to 50-s mindful movement practice video] [link to 12-min mindful walking audio]	This is a hard moment. Send kindness to yourself for what you are experiencing right now. Here are a few practices that could help [link to “may I be well” image] [link to 6 ways to practice self-compassion video]

### JIT Messages and the Ecological Momentary Assessments That Trigger Them

To provide support to adolescents in moments and contexts of high need, ecological momentary assessment (EMA) messages are sent to participants twice a day (first thing in the morning, and right before bed) to assess levels of stress and mindfulness. When respondents endorse relatively low levels of mindfulness [i.e., >30 on a scale from 1 (very mindLESS) to 100 (very mindFUL)] or high levels of stress [i.e., >70 on a scale from 0 (not stressed at all) to 100 (very stressed)], they are sent brief messages to acknowledge that they seem to be experiencing a challenging moment, and to provide a brief suggestion about ways to increase mindfulness in times of need. In addition, accompanying each brief message are direct links to two relevant practices in the on-demand library (see Table 2 for example JIT messages).

### Dissemination of Product

These materials are available upon request from the first author.

## Discussion

Our goal in this paper was to describe the development and content of a multi-method, adaptive supplement to an evidence-based MBI for adolescents. We have argued here and elsewhere (15) that it is critical to investigate supplements such as these to in-person, group mindfulness programs to better support skill transfer and the establishment of a regular mindfulness practice, particularly in adolescence. This assertion is based on evidence that MBIs are well-liked by and effective for adolescents (5–12), but effect sizes for MBIs are small-to-moderate and variable in size (5, 18), and compliance rates with home practice recommendations in adolescence are very poor (22).

The first step in investigating technological supplements to in-person mindfulness programs is the creation of a supplement that can augment and support what participants learn in the group program. We have aimed to do that with L2B Plus, described here, with multiple methods to support developing a formal mindfulness practice, applying mindfulness in daily life, and remaining mindful during periods of stress. The creation of this multi-method supplement is therefore a critical next step in the science of the implementation of MBIs. In future research, we intend to test the extent to which L2B Plus is a feasible and acceptable intervention approach for adolescents (48), explore whether L2B Plus seems effective to reduce adolescent stress and anxiety (48), identify the particular elements of the multiple Plus components that are the most effective at improving adolescent outcomes, and determine the extent to which there is specific added value in the Plus components over and above the in-person, group L2B program. Evidence that L2B Plus is feasible and acceptable, and that the multi-method supplement improves adolescent outcomes over and above the group program, would also suggest that clinical applications of L2B might benefit from incorporation of Plus components. Randomized controlled trials will be important to evaluate internal validity; in addition, community-based work with diverse samples will contribute critical information about generalizability.

Although originally developed for use in high school settings, L2B has been expanded to community settings (12) and also for use at different ages, including middle school and college aged students, as well as residents in senior living facilities. We believe that L2B Plus has great potential to support the development of a mindfulness practice in any setting, context, and age range that L2B is applied to, but it will be important to evaluate participant- and setting-level characteristics that increase or decrease feasibility, acceptability, and effectiveness of L2B Plus. For instance, because L2B Plus incorporates a multi-method supplement that is technologically based, individuals who do not have access to or are not comfortable with technology may experience barriers to engaging with L2B Plus. However, the broader literature on EMIs has dealt with these issues, and also provides evidence that even individuals uncomfortable with technology can benefit from EMIs, particularly with additional attempts to reduce fear of and increase comfort with technology (27). Although access to technology to support L2B Plus is another potential obstacle to its successful implementation, mobile phone, and/or tablet ownership is relatively equally distributed across racial/ethnic and socioeconomic groups, including in adolescence (23, 24). Therefore, EMI supplements typically do not create health disparities because of inequitable patterns of access to mobile technology, but instead may actually be a way to more equitably deliver treatment. In addition, this issue is also one that applies to the broader use of EMIs, and this broader work provides evidence-based solutions to problems with access to technology (e.g., providing mobile phone access to participants) (27). The L2B Plus approach, particularly with possibilities for self-tailoring that are built in, is in line with trends toward personalized prevention. By allowing choice and acknowledgment of unique needs for adolescents, this supplement is in keeping with personalized intervention methods to promote engagement and practice.

Our goal is to increase the efficacy of MBIs targeting adolescents through the development and, in the future, integration of technological support for skill transfer from an in-person MBI to daily life. It will be critical in future work to empirically evaluate the benefit of the multi-method adaptive supplement that we have described here, but its development is a crucial next step in the implementation of interventions to increase adolescent mindfulness.

## Data Availability Statement

The raw data supporting the conclusions of this article will be made available by the authors, without undue reservation.

## Ethics Statement

The studies involving human participants were reviewed and approved by Colorado State University Institutional Review Board. Written informed consent was obtained from parents as well as adolescents 17 years of age and older; written informed assent was obtained from all other adolescents.

## Author Contributions

RL-T, SR, and NS wrote the initial draft of the manuscript. PB, JS, JC, and KH read and revised the manuscript. All authors were involved with study design, supplement development, and approved the publication of this manuscript.

## Conflict of Interest

PB receives a royalty from Learning 2 BREATHE when the manual is purchased. The remaining authors declare that the research was conducted in the absence of any commercial or financial relationships that could be construed as a potential conflict of interest.
